# Supplementary material for: Design, Synthesis, and Potent Anticancer Activity of Novel Indole-Based Bcl-2 Inhibitors
Source: Int J Mol Sci. 2023 Sep 28;24(19):14656. doi: 10.3390/ijms241914656 (PMC10572575; doi:10.3390/ijms241914656)

Supplementary figures

Supplementary Figure 1. [1] HNMR of U2.

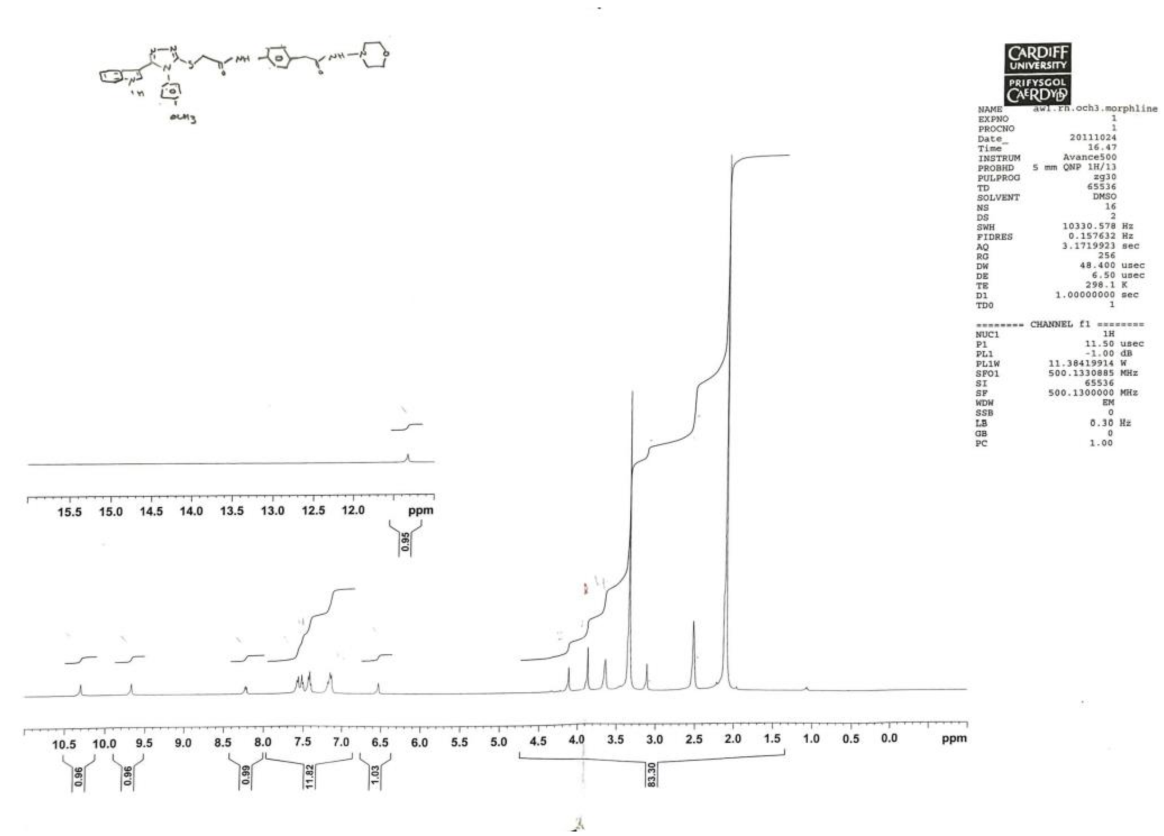

Supplementary Figure 2. ^{13}**CNMR of U2.**

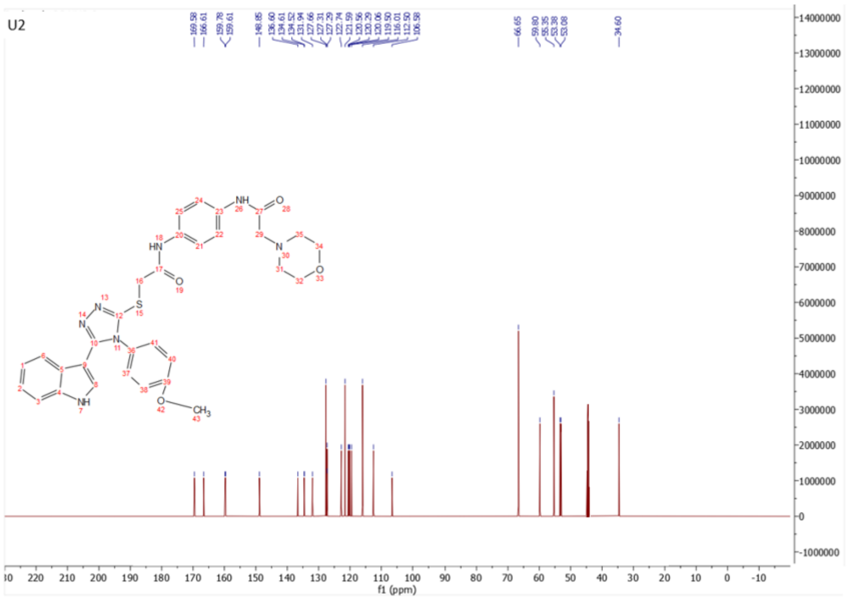

Supplementary Figure 3. [1] **HNMR of U3.**

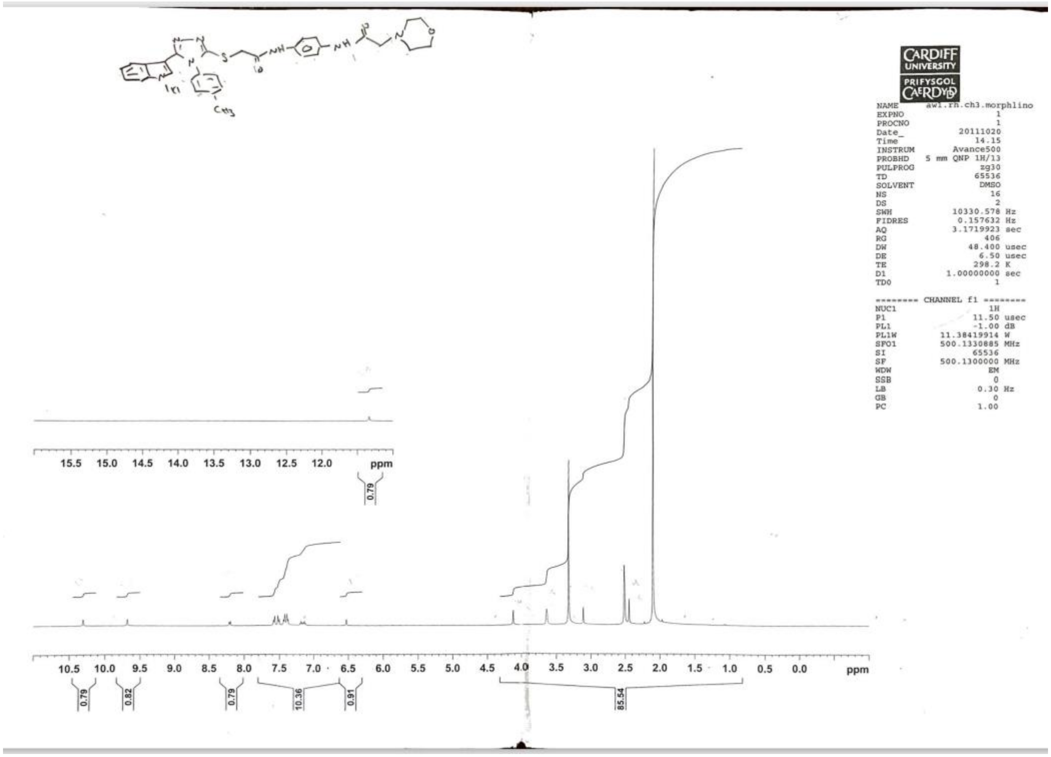

Supplementary Figure 4. ^{13}CNMR of U3.

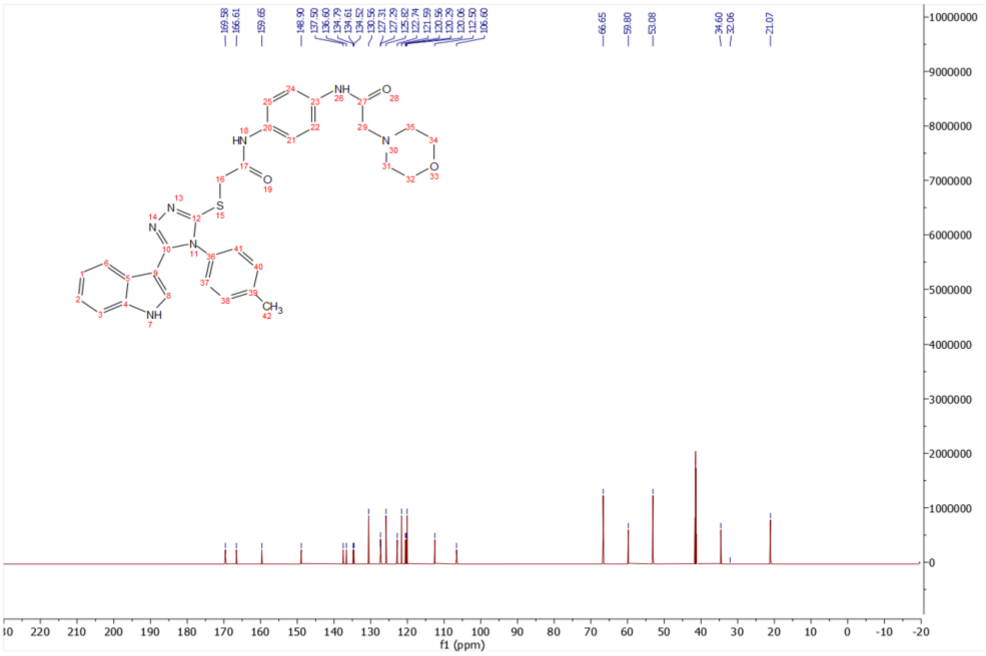

Supplement: Supplementary file 1 [file ijms-24-14656-s001.zip › ijms-2552609-supplementary.pdf]
